# Algorithmic assessment of missense mutation severity in the Von-Hippel Lindau protein

**DOI:** 10.1371/journal.pone.0234100

**Published:** 2020-11-05

**Authors:** Francisco R. Fields, Niraja Suresh, Morgan Hiller, Stefan D. Freed, Kasturi Haldar, Shaun W. Lee

**Affiliations:** 1 Boler-Parseghian Center for Rare and Neglected Diseases, University of Notre Dame, Notre Dame, Indiana, United States of America; 2 Department of Biological Sciences, University of Notre Dame, Notre Dame, Indiana, United States of America; 3 Chemistry-Biology-Biochemistry Interfaces, University of Notre Dame, Notre Dame, Indiana, United States of America; 4 Eck Institute for Global Health, University of Notre Dame, Notre Dame, Indiana, United States of America; Lund University, SWEDEN

## Abstract

Von Hippel-Lindau disease (VHL) is an autosomal dominant rare disease that causes the formation of angiogenic tumors. When functional, pVHL acts as an E3 ubiquitin ligase that negatively regulates hypoxia inducible factor (HIF). Genetic mutations that perturb the structure of pVHL result in dysregulation of HIF, causing a wide array of tumor pathologies including retinal angioma, pheochromocytoma, central nervous system hemangioblastoma, and clear cell renal carcinoma. These VHL-related cancers occur throughout the lifetime of the patient, requiring frequent intervention procedures, such as surgery, to remove the tumors. Although VHL is classified as a rare disease (1 in 39,000 to 1 in 91,000 affected) there is a large heterogeneity in genetic mutations listed for observed pathologies. Understanding how these specific mutations correlate with the myriad of observed pathologies for VHL could provide clinicians insight into the potential severity and onset of disease. Using a select set of 285 ClinVar mutations in VHL, we developed a multiparametric scoring algorithm to evaluate the overall clinical severity of missense mutations in pVHL. The mutations were assessed according to eight weighted parameters as a comprehensive evaluation of protein misfolding and malfunction. Higher mutation scores were strongly associated with pathogenicity. Our approach establishes a novel *in silico* method by which VHL-specific mutations can be assessed for their severity and effect on the biophysical functions of the VHL protein.

## Introduction

Von Hippel-Lindau (VHL) disease is an autosomal-dominant hereditary disease associated with the development of multiple angiogenic tumor types. This includes clear cell renal carcinoma (ccRCC), retinal angioma (RA), central nervous system hemangioblastoma (CHB), and pheochromocytoma (PCC) [1,2]. The presence or absence of PCC divides VHL disease into type 1 or type 2. Type 2 VHL is further subdivided into three subtypes depending on the appearance of other cancers: type 2A, PCCs but no ccRCCs, type 2B, PCCs and ccRCCs, or type 2C, PCCs only [1]. While this allows for some preliminary genotype-phenotype associations, a patient’s association with a specific subtype alternates as different cancers arise throughout their lifetime [1].

Patients with VHL disease have a single mutation in one allele of the *VHL* gene [3]. Upon spontaneous inactivation of the second allele, tumor development can initiate, making the loss of heterozygosity (LOH) a crucial step in the development of VHL disease [1,4,5]. The *VHL* gene encodes two protein products, both of which exhibit equivalent activity: the 30kDa isoform (pVHL_30_) and the more common 19kDa isoform (pVHL_19_) found in most tissues [6,7]. pVHL forms a complex with elongin B (EloB) and elongin C (EloC) for the VCB complex [8–10]. This stabilizes EloB, EloC, and pVHL, making them resistant to proteosomal degradation; however, upon mutation of pVHL, contacts with EloB and C become disrupted, making pVHL unstable and a target for degradation [9,11,12]. VCB then complexes with cullin 2 (Cul2) and the RING finger protein RBX1 to form the VCB-CR complex [9]. This complex functions as an E3 ubiquitin ligase, targeting a variety of proteins for degradation by the proteasome [13–15].

Both HIF-1α and HIF-2α are ubiquitinated by the VCB-CR complex for degradation by the proteasome, since both share a similar binding site to pVHL [2,13,16,17]. HIF is involved in cellular oxygen sensing and regulates the expression of angiogenic genes making it a key player in the development of the vascularized tumor pathologies associated with VHL disease [16,18]. Under normoxic conditons, HIF is hydroxylated on two proline residues allowing for interaction with pVHL and its subsequent ubiquitination by the VCB-CR complex [2,16]. In hypoxic conditions, HIF is not hydroxylated, preventing negative regulation by pVHL. Under these conditions, active HIF subsequently drives the expression of hypoxia associated genes. Loss of functional pVHL allows aberrant expression of HIF target genes, such as vascular endothelial growth factor, contributing to the development of VHL associated angiogenic tumors [19–21].

Regardless of VHL subtype, patients are at a lifetime risk for the development of tumors with the age of onset of VHL disease ranging from 20 to 40 years old [22]. Clinical diagnosis of VHL disease is dependent upon the familial history of VHL. Patients with a family history of VHL must present with CHB, PCC, or ccRCC; however, if there is no family history of disease, patients must then present with two more CHBs or a CHB and a visceral tumour, such as ccRCC [1,2,22]. Genetic testing is conducted for presymptomatic detection of VHL for patients with a family history of disease [23]. Surveillance, which varies since there are many tissue types in which the VHL tumors and cysts can arise, includes ophthalmologic evaluation and CT or MRI scans [22,24]. Similar to surveillance, treatment is also varied due to the breadth of tumor types and includes surgery, radiation, or chemotherapies [22,24].

Multiple studies have investigated the association of mutation types to the VHL subtypes; however, there is still heterogeneity associated with the phenotypes of missense mutations [25–27]. While loss-of-function mutations cause global disruption of the VHL protein, missense mutations may only affect certain interaction partners and cellular pathways involving pVHL [28]. A recent study completed by Razafinjatovo et al used an *in silico* approach to determine the thermodynamic stability of a given pVHL mutation [29]. It was determined that the most thermodynamically unstable missense mutations resulted in pathogenic disease via global destabilization of pVHL and stabilization of HIF. This suggests that while some VHL missense mutations might only affect specific functions of the protein, others cause global misfolding and destabilization of the protein. A comprehensive examination of the effects of a given missense mutation for pVHL can provide significant insight into how a given patient mutation can be predictive of disease severity; however, a systematic examination of the role of a given missense mutation (and subsequent amino acid replacement) must take into account multiple factors: secondary structure, thermodynamic stability, binding partners, translation rate, among other biophysical and biochemical properties. Providing a predictive scale of the phenotypic severity of a given missense mutation using *in silico* evaluation can potentially inform clinicians to develop tailored screening and surveillance strategies for each patient. Currently, some publically available online databases provide investigators with basic information on the pathogenicity of a given mutation in genetic diseases, including VHL. *ClinVar* provides basic annotation on the pathogenicity of curated mutations according to the American College of Medical Genetics and Genomics (ACMG) [30,31]. These guidelines provide a spectrum of pathogenicity descriptors for mendelian genetic diseases. Within these guidelines, mutations annotated as “pathogenic” or “likely pathogenic” have a greater than 90% certainty of a given gene variant being disease causing [31]. Leveraging these sources of phenotypic information can help train and refine predictive algorithms for the assessment of missense mutation severity. Previously, we developed a computational, multiparameteric approach to evaluate the biophysical consequences of missense mutations on the structure and stability of the Mucopolysaccharidosis Type IIIA (Sanfilippo Syndrome) protein (MPSIIIA). Severe mutations identified through our scoring approach correlated to a higher clinical severity of Sanfilippo Syndrome [32]. We observed that mutations more deleterious to overall enzyme folding and function were correlated to more severe disease outcomes and a higher multiparameteric algorithm scores [32]. In this study, we created an advanced weighted-score multiparametric approach to validate the use of a computational algorithm to assess the potential disease severity of genetic missense mutations in pVHL. We focused not only on mutations that can affect the overall proteostasis of pVHL, but also noted the specific mutations that would impact VHL-specific functional properties [28,29,33]. Our multiparametric algorithm for VHL included a set of eight biophysical parameters with individually weighted scores that gave an overall assessment of the ability of a given missense mutation in VHL to result in protein impairment: 1. aggregation propensity; 2. protein-protein interactions; 3. secondary structure; 4. conformational flexibility; 5. solvent accessibility; 6. protein stability; 7. post-translational modifications, and 8. translational rate [9,14,18,32].

## Materials and methods

### Mutation sets

A set of 285 missense mutations in the human VHL gene, arising from a single nucleotide polymorphism (SNP) was acquired from ClinVar (https://www.ncbi.nlm.nih.gov/clinvar/) [30] (S2 File). An additional set of 1380 mutations was generated to represent all possible theoretical missense mutations (APMM) of VHL from a SNP (S1 File). Finally, hot spot mutations and mutation lists associated with different pathogenic outcomes were selected from the literature [26,29,33–35] (S3 File). A total of 1665 mutations were therefore used in our multiparametric analysis.

### Structures used in analysis

The VHL crystal structure in complex with EloB, EloC, and Cul2 was used in Parameters 2, 3, 5, and 6 (1VCB) [36]. Crystal structures in complex with HIF-1a were also used to develop Parameter 2 (1LM8, 4WQO) [9,37]. The unstructured N-terminus of VHL is missing from published crystal structures; therefore, to assess the effect of mutations in this region for their effects on protein stability, ITASSER (https://zhanglab.ccmb.med.umich.edu/I-TASSER/) was used to generate a putative structure of VHL as input for Parameter 6 [38].

### Parameters for algorithmic assessment

#### Parameter 1: Aggregation propensity

Aggregation propensity was calculated as previously described [32]. A positive aggregation score was assigned if a given mutation enhanced the hydrophobic character and aggregation propensity of the VHL polypeptide chain. *AGGRESCAN* (http://bioinf.uab.es/aggrescan/) was used to assess the individual contributions of an amino acid change on the overall hydrophobicity and propensity for aggregation [39].

#### Parameter 2: Protein-protein interactions

VHL functions as an E3 ubiquitin ligase when bound to HIF, EloB, EloC, and Cul2 [9,10,19,40]. To assess the capacity of missense mutation to disrupt these crucial interactions, mutations occurring at positions found to mediate protein interactions with known binding partners were scored positive.

#### Parameter 3: Secondary structure

VHL consists of three structural domains: an N-terminal random coil region, a beta sheet containing β-domain, and an alpha helical α-domain. Maintaining the secondary structural elements in this region are crucial for pVHL function as pathogenic mutations are less likely to occur in other disordered regions of the protein [29,33]. If a missense mutation occurred in a region of secondary structure, it was scored positive for this parameter.

#### Parameter 4: Conformational flexibility

Flexibility allows a protein molecule to perform its function and bind to substrates and interaction partners [41]. The overall flexibility of a given protein is governed by the location of key amino acids within the amino acid sequence. The unique conformational constraint of the proline side chain and the ability to accommodate a *cis-/ trans*-conformation in proteins makes proline a significant contributor to overall protein flexibility and function [42,43]. Glycine residues contain a side chain that prevents steric hindrance, increasing the flexibility of a protein [44,45]. Finally, cysteine residues are capable of disulfide bonds, which are crucial components of protein stability [46,47]. In this analysis, any missense mutation involving changes in proline, glycine, or cysteine residues were scored as positive.

#### Parameter 5: Solvent accessibility

Replacing surface exposed hydrophilic residues with hydrophobic residues or charged residues with uncharged residues and vice versa can increase the probability of effects on protein-protein interaction and overall protein aggregation [47]. In addition, substitution of hydrophobic amino acids for hydrophilic ones within the core of the protein can be thermodynamically unfavorable [48]. Finally, the position of charged residues within the protein can be crucial for intramolecular salt bridge formation. Deleterious mutations could destabilize these interactions, thereby destabilizing the protein [49]. If a mutation reversed or removed a charge at a given position, replaced a buried hydrophobic residue with a hydrophilic residue, or resulted in a surface exposed hydrophilic residue becoming hydrophobic, it was scored as positive in this parameter.

#### Parameter 6: Protein stability

Proteins have evolved to fold into specific structures in order to perform their roles in the crowded environment of the cell. We evaluated the effects of missense mutations on the stability of pVHL as destabilizing mutations could prevent proper folding and function. *In vivo* protein folding relies on both thermodynamic and kinetic stability [50,51]. The difference in the energy states of the unfolded and that native protein is the thermodynamic stability while kinetic stability refers to the energy barriers that separate any two states of a protein [50,52–55]. A missense mutation can alter both the thermodynamic and kinetic stability of a protein indicating a biophysical cause for disease. To determine if overall protein stability was altered via a missense mutation, pVHL missense mutations were assessed using the CUPSAT (http://cupsat.tu-bs.de/) online prediction server [50]. Missense mutations that resulted in a -ΔΔG, i.e., indicating significant changes in overall protein stability, were scored as positive [50].

#### Parameter 7: Post-translational Modifications (PTMs)

Post-translational modifications serve crucial roles on proteins through the covalent addition of small molecules to protein backbones [56]. PTMs confer additional specificity to the overall structure and function of a given protein, and contribute to the ability of a protein to interact with different binding partners [56–58]. To assess the specific roles of PTMs in our algorithmic assessment of VHL disease, missense mutations that occurred at a position known to be post-translationally modified were positively scored [33].

#### Parameter 8: Translation rate

A change in the translation rate of a protein can have deleterious effects on folding [59,60]. Translation rated is dependent on the codon usage percentage and the number of rare versus common codons in the gene and the subsequent abundance of the corresponding tRNA species. A mutation was scored in this parameter if the mutation change resulted in a translation rate fold change exceeding +2 or -2. Translation rate was calculated using the codon usage tables and tRNA abundances at GtRNAdb (http://gtrnadb.ucsc.edu/) [61].

### Overall score

The overall score given to the multiparametric assessment of each gene mutation was calculated as a sum of the unweighted or weighted scores as described previously [32].

### Parameter independence and weighting strategy

Parameters were tested for independence from one another using Spearmans rho correlation in R. Parameters with rho values < .5 and > -.5 were considered not correlated (S1 Table). To determine an optimized strategy for weighting score values for each of the parameters, 211 ClinVar mutations were used with their corresponding pathogenicity indicators to develop a pathogenicity index. ClinVar mutations annotated as benign, uncertain significance, or conflicting interpretations were considered “benign” and given a pathogenicity score of 0. Those annotated as likely pathogenic or pathogenic were considered “pathogenic” and given a score of 2. Symphony (http://biosig.unimelb.edu.au/symphony/), an online program to predict the risk of ccRCC in a given VHL mutation, was also used to develop the pathogenicity index by scoring the same 211 ClinVar mutations. Mutations identified as high risk of ccRCC were given a score of 1 while those identified as low risk were given a score of 0. The scores were summed for each mutation, creating a pathogenicity index ranging for 0 to 3 for each of the 211 ClinVar mutations. A chi-square was used to test for dependence of the pathogenicity index score to the unweighted scores of each parameter using R. The resulting p-values were used to set the following cut-offs for our weighting approach. P < .005 was weighted 4. .005 < P < .05 was weighted 3. .05 < P < .5 was weighted 2. Finally, P > .5 was unweighted (i.e. score of 1) (S2 Table).

### Statistical analysis

All statistical analysis was conducted using GraphPad Prizm (S4 File). Spearmans rho and Chi-square tests were performed in R (S1 and S2 Tables).

## Results

Using a set of 285 missense mutations from the ClinVar database and another set of 1380 possible missense mutations (APMM) in pVHL, we began to evaluate the consequences of missense mutations, arising from a SNP. Our multiparametric approach provided a holistic view of the consequences of a mutation on the overall structure and stability of pVHL by evaluating the following parameters: aggregation propensity, protein-protein interactions, secondary structure, conformational flexibility, solvent accessibility, protein stability, post-translational modification, and translational rate.

### Unweighted scores for all possible mutations and the ClinVar dataset

Using our initial, unweighted approach, in which a scored mutation received a 1 and an unscored mutation received a 0, we obtained a range of values for all missense mutation from 0 to 7 for both the APMM and the ClinVar data sets, indicating no single mutation received a score in all of the 8 parameters (S1 Fig). Using the ClinVar data set, all of the parameters were determined to be independent of one another (S1 Table). The average score of the APMM and the ClinVar data sets were 2.7 and 2.8, respectively (S4 File). Our unweighted approach did not result in significant separations in the benign and pathogenic mutations (means of 2.46 and 3.59, respectively); therefore, we next evaluated the scores using a weighted approach (S2A Fig and S4 File).

### Weighted scoring approach

**Fig 1:**
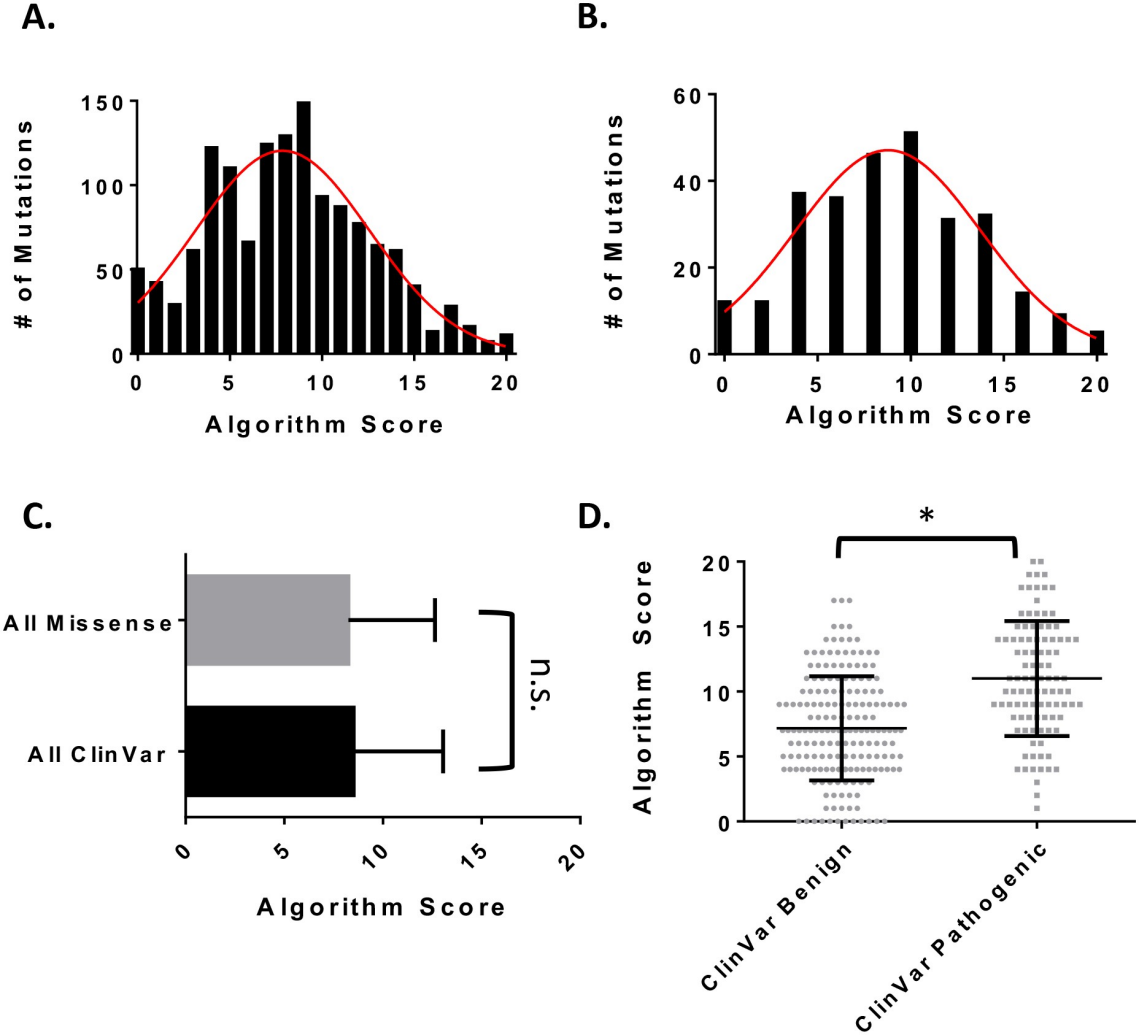
Score distributions for the VHL missense mutations used in the multiparametric approach. **A.** A fitted Gaussian distribution (red) of scores for all 1379 possible missense mutations from a SNP in VHL **B.** A fitted Gaussian distribution (red) of scores for the 285 ClinVar missense mutations used in this study. **C.** Relationship between the All Mutation data set and the ClinVar data set. **D.** Mutation algorithm scores plotted according to their ClinVar pathogenicity. Each dot is a mutation. All error bars represent the standard deviation. A * represents a P < .05 according to a Kolmogorov Smirnov test. All statistics done in Graph Pad Prizm.

In order to improve our strategy for the algorithmic assessment of missense mutations, a weighting strategy was developed using the pathogenicity indications available on ClinVar and Symphony, an online predictor of ccRCC risk of mutations in VHL. This pathogenicity index was tested for dependence against the unweighted parameters using the chi-square statistic. Weights were then assigned to the parameters according to their resulting p-value (S2 Table). This new scoring approach resulted in a range of scores from 0 to 20 for both the APMM sand the ClinVar data sets with means of 8.3 and 8.5, respectively (Fig 1A and 1B and S4 File). These populations were not found to be significantly different from one another by a Kolmogorov Smirnov test (P = .91) (Fig 1C). Upon comparing the benign ClinVar mutations to the pathogenic ClinVar mutations, we observed a significant shift in the mean score from 7.2 to 11.0 respectively (S2B Fig and S4 File). This was determined to a be significant difference according to a t-test with a p < .05 (Fig 1D). This ClinVar set was further subdivided into its original ClinVar pathogenicity indications. All of the pathogenic groups (likely pathogenic, likely pathogenic/pathogenic, and pathogenic annotation) showed significant separation from the mutations of uncertain significance (S3A Fig). Symphony was also used to determine the risk of ccRCC associated with the ClinVar mutations used in our pathogenicity index. When comparing the risk of ccRCC for the ClinVar mutations, we observed a significant difference in the algorithm score between the mutations identified as high risk (mean score of 10.5) and those identified as low risk (mean score of 7.6) (S3B Fig). Our approach to refine the weights of each parameter therefore, was successful in distinguishing populations of pathogenic mutations and benign mutations from those databases listed.

### Algorithm scores according to location within VHL 3-D structure

**Fig 2:**
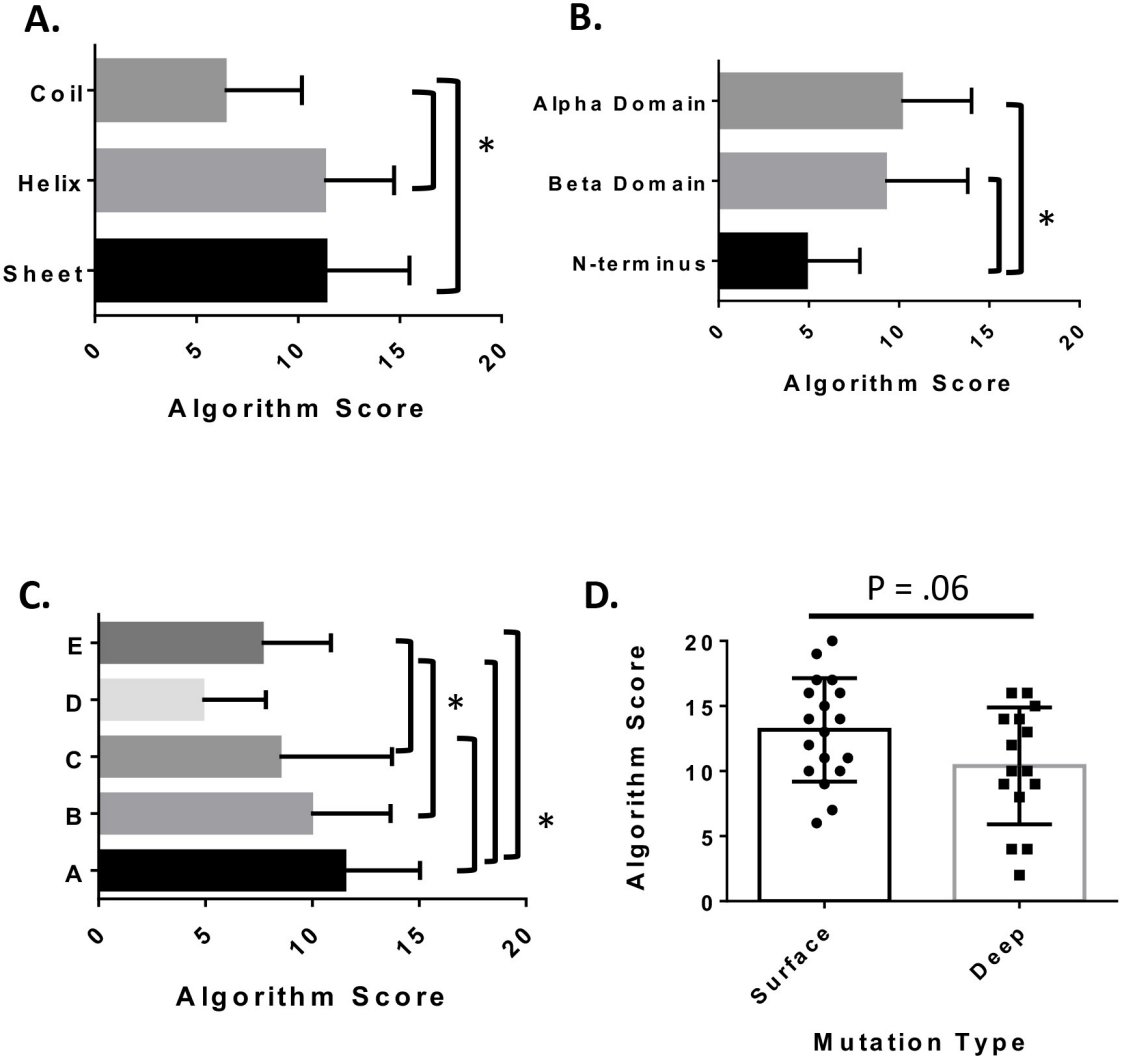
Association of missense mutation algorithm score to its spatial distribution on pVHL. **A.** Algorithm scores for mutations according to secondary structure. **B.** pVHL domain **C.** or pVHL binding interfaces. Significance was determined using an ANOVA or Kruskal-Wallis test and followed up with Tukey HSD or Dunn’s MCT as appropriate. Error bars represent the standard deviation. * represents a significant difference with a p < .05. **D.** Algorithm Score for mutations according to their depth within the structure of VHL. Each dot is a mutation. Error bars represent the standard deviation. * represents a significant difference with a p < .05 as determined by Student’s t-test. All statistics were done using GraphPad Prizm.

VHL consists of three structural domains: an N-terminal random coil region, a beta sheet containing β-domain, and an alpha helical α-domain. Pathogenic mutations have been observed to occur at a lower frequency in areas of disorder; therefore, maintaining this arrangement of secondary structure motifs is predicted to be critical for functional pVHL [29,33]. We therefore predicted that we should also observe higher algorithm scores in mutations that occur in areas of defined secondary structure. Indeed, mean algorithm scores were significantly higher in regions of helix or sheet compared to random coil regions of the VHL protein (Fig 2A). Overall secondary structure dictates the division of pVHL into three main domains: the α-domain, the β-domain, and the N-terminal coil region [33]. We determined that mutations scored higher if they occurred in the α and β domains compared to the N-terminus (Fig 2B).

VHL also consists of five binding interfaces [33,62,63]. Interface A is involved in VCB complex formation [9]. The HIF-1α binding site is located within interface B [37,40]. Cul2 interacts with interface C [33,40]. The unstructured N-terminus of VHL is proposed as interface D, though little is known of its binding partners and their importance in the progression of VHL disease [8,33]. However, there are residues in interface D that are candidates for phosphorylation by aurora kinase II and casein kinase II [8,33]. Finally, interface E, consisting of the helical C-terminus, is predicted to interact with Zinc-finger protein 197 (ZNF-197) and Von-Hippel Lindau Binding Protein 1 (VBP1), a protein chaperone [8,33,64]. Due to the importance of each VHL protein interface (A,B,C) in the ubiquitin ligase function of VHL and subsequent HIF regulation by VHL, we expected to observe higher average algorithm scores for mutations occurring within these interfaces [2,40,65,66]. When the mean algorithm scores for each of the binding interfaces were compared, we observed significantly higher scores within interface A compared to interfaces C, D, and E (Fig 2C). Interfaces B and C, important binding surfaces for HIF and Cul2 respectively, also had significantly higher algorithm scores than interfaces D and E, which are not involved in VCB complex formation (Fig 2C).

Recent studies into the distribution of mutations within the VHL structure have observed that amino acid changes occurring on the surface of the pVHL are more deleterious for overall function [33,34]. These corresponding deleterious genetic mutations are associated with a higher risk of pheochromocytoma (PCC), a cancer of the adrenal glands that causes hormone dysregulation [2,33,34]. To determine if these mutations are detected by our algorithmic scoring method, we compared the average algorithm score for mutations at the protein surface and mutations in the protein core [34]. Since VHL functions as a scaffold for the assembly of the VCB complex, we would expect that mutations occurring on the surface of the protein, and therefore affecting the binding sites for interaction partners, would result in higher algorithm scores and more severe disease. The p-value (p = .06) indicated that the algorithm scores for comparing the surface versus core-located mutations approached significance at the .05 α-value, suggesting that the observed trend towards a higher algorithm score (mean score = 13.2) in the surface mutations versus mutations occurring deeper in the VHL structure (mean score = 10.4) may have biological importance (Fig 2D). However, additional data and sampling of mutations appropriate for these regional comparisons are needed to improve the statistical score.

### Identification of highly destabilizing and hot spot VHL mutations with algorithmic assessment

We investigated the capacity of our algorithm to identify mutations that have been described as highly destabilizing to VHL [29]. Razafinjatovo et al identified W117 and L184 as missense mutation hotspots that can highly destabilize pVHL [29]. Our multiparameteric algorithm approach also scored mutations at theses residues considerably higher than the pathogenic mean score of 11 (mean score for APMM at W117 = 12.86 and mean score for APMM at L184 = 14.83), both above the average score for the pathogenic ClinVar mutations (Table 1). Other VHL mutation hot spots, such as L158 and N78 (scores of 13.0 and 16.3, respectively), also scored highly above the average for pathogenic ClinVar mutations; however, R167, another annotated VHL hotspot, received a below average pathogenic score (9.6) (Table 2). Finally, other hotspot mutations, such as Y98 (mean score of 6.8), scored below average for benign scores. Our multiparametric scoring algorithm is designed to provide an evaluative sum of how a given missense mutation will affect the ability of a protein to fold and function properly. In this way, pathogenic mutations such as Y98, with low algorithm scores may not ultimately cause disease phenotypes by destabilizing pVHL protein, but through a more direct local effect that is critical for VHL function and protein interaction. This is likely the case for Y98, located in binding interface B, which is crucial for interaction with HIFα [40,67]. Therefore, mutations at these positions (Y98 mutations all score in parameter 2) are sufficient to cause disease through their ability to uniquely affect protein-protein interactions (Table 2). Other studies have found that specific mutations at the Y98 position will cause different VHL cancer phenotypes with Y98H causing type 2B disease and Y98N causing type 2A disease by modulating the efficacy of binding to HIFα [19]. Although these kinds of critical mutations (crucial binding site, catalytic abatement, posttranslational substrate) should be taken into account independently from our algorithm, the use of our algorithm scores *combined* with these additional considerations will serve as a valuable comprehensive evaluation for the protein.

**Table 1:** All possible missense mutations at highly destabilizing residues and their corresponding algorithm scores.

Algorithm Scores for All Possible Missense Mutations at Highly Destabilizing Residues											
Mutation	P1: Aggregation Propensity	P2: Protein Protein Interactions	P3: Secondary Structure	P4: Conformational Flexibility	P5: Solvent Accessibilty	P6: Protein Stability	P7: Post-translational Modifications	P8: Translation Rate	Total Score	Average Score	Standard Deviation
**W117R**	0	4	4	0	3	0	0	0	11	12.86	1.77
**W117R**	0	4	4	0	3	0	0	0	11		
**W117G**	0	4	4	0	0	4	0	0	12		
**W117S**	0	4	4	0	0	4	0	0	12		
**W117L**	2	4	4	0	0	4	0	0	14		
**W117C**	0	4	4	2	0	4	0	1	15		
**W117C**	0	4	4	2	0	4	0	1	15		
**L184I**	0	4	4	0	0	4	0	1	13	14.83	2.32
**L184V**	0	4	4	0	0	4	0	0	12		
**L184F**	0	4	4	2	0	4	0	0	14		
**L184H**	0	4	4	0	3	4	0	0	15		
**L184P**	0	4	4	2	3	4	0	1	18		
**L184R**	0	4	4	2	3	4	0	0	17		

**Table 2:** All possible missense mutations at VHL disease associated mutation hot sports and their corresponding algorithm scores.

Algorithm Scores for All Possible Missense Mutations at VHL Mutation Hot Spots											
Mutation	P1: Aggregation Propensity	P2: Protein Protein Interactions	P3: Secondary Structure	P4: Conformational Flexibility	P5: Solvent Accessibilty	P6: Protein Stability	P7: Post-translational Modifications	P8: Translation Rate	Total Score	Average Hot Spot Score	Standard Deviation
**R167W**	0	0	4	0	3	0	0	0	7	9.60	1.95
**R167L**	2	0	4	0	3	0	0	0	9		
**R167P**	0	0	4	2	3	0	0	0	9		
**R167G**	0	0	4	0	3	4	0	1	12		
**R167Q**	0	0	4	0	3	4	0	0	11		
**L158Q**	0	4	4	0	0	0	0	1	9	13.00	3.24
**L158R**	0	4	4	2	3	0	0	0	13		
**L158M**	0	4	4	0	0	4	0	0	12		
**L158V**	0	4	4	0	0	4	0	1	13		
**L158P**	0	4	4	2	3	4	0	1	18		
**N78S**	0	4	4	0	0	4	0	0	12	16	2.55
**N78T**	0	4	4	0	0	4	0	1	13		
**N78D**	0	4	4	0	3	4	0	1	16		
**N78H**	0	4	4	0	3	4	0	1	16		
**N78I**	2	4	4	0	3	4	0	1	18		
**N78K**	0	4	4	2	3	4	0	1	18		
**N78K**	0	4	4	2	3	4	0	1	18		
**N78Y**	2	4	4	2	3	4	0	0	19		
**Y98S**	0	4	0	0	0	0	0	1	5	6.83	0.98
**Y98C**	0	4	0	2	0	0	0	1	7		
**Y98D**	0	4	0	0	3	0	0	0	7		
**Y98H**	0	4	0	0	3	0	0	0	7		
**Y98N**	0	4	0	0	3	0	0	0	7		
**Y98F**	0	4	0	0	0	4	0	0	8		

### VHL missense mutations score and onset of VHL related cancers

**Fig 3:**
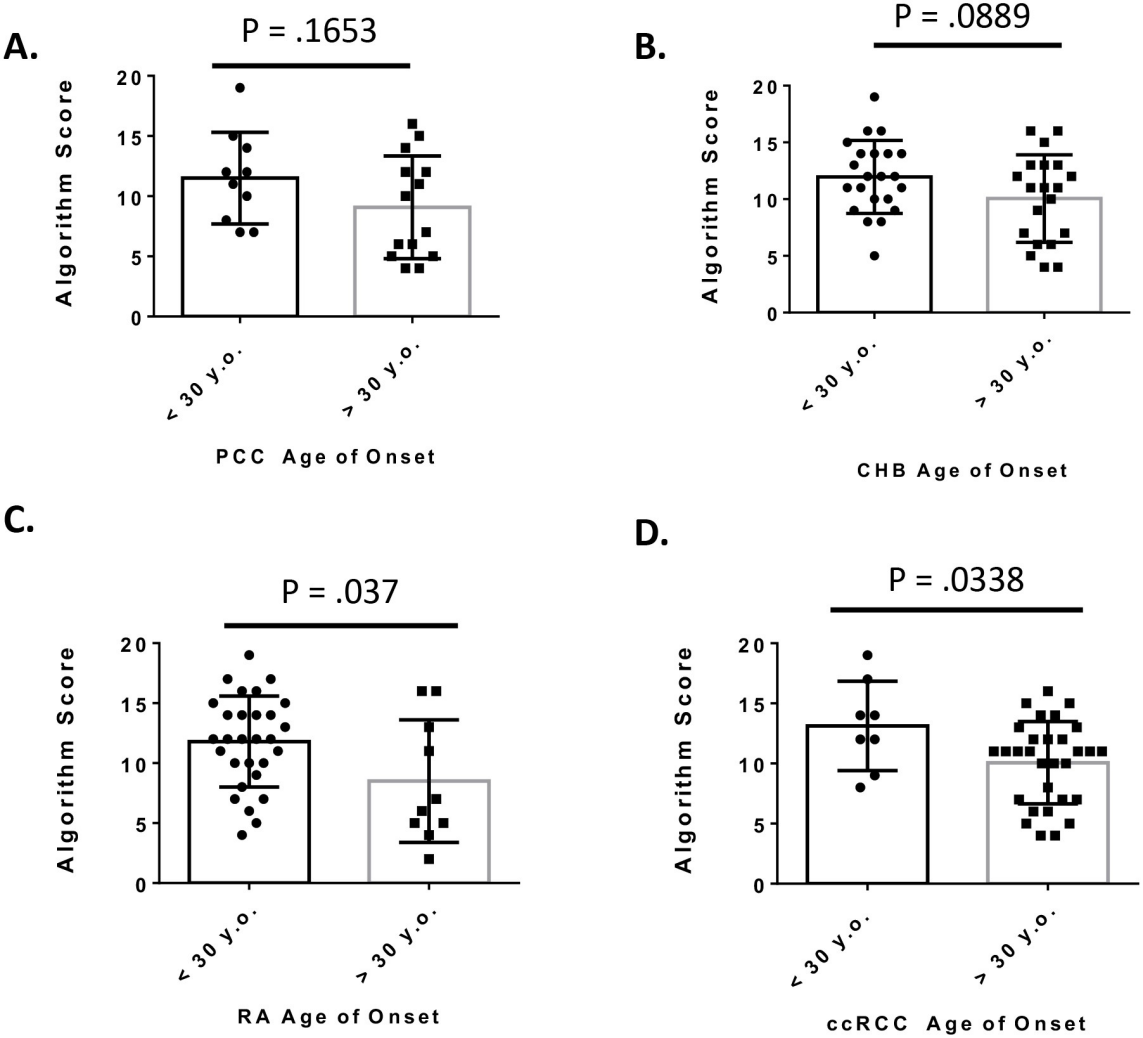
VHL missense mutations algorithm scores associated with onset of the VHL related cancers: **A.** pheochromocytoma (PCC) **B.** central nervous system hemangioblastoma (CHB) **C.** retinal angioma (RA) and **D.** clear cell renal carcinoma (ccRCC). Each dot is the average age of onset for a missense mutation. Error bars represent the standard deviation. P-values were determined using Student’s t-test.

Next, we assessed if our algorithm would be able to identify missense mutations that are more likely to be associated with an early age of onset of VHL-related pathologies. Using published data sets of missense mutations from Chinese patients (available in Peng et al) and another dataset of English patients (available in Ong et al), we compared the algorithm score for 56 missense mutations in early (less than 30 years old) to late (greater than 30 years old) onset of pheochromocytoma (PCC), central nervous system hemangioblastoma (CHB), retinal angioma (RA), and clear cell renal carcinoma (ccRCC) [26,34]. For PCC, we observed a shift towards a higher average algorithm score for mutations associated with an early age of onset (11.5) than mutations with a later age of onset (9.1); however, this difference was not statistically significant (p = .1653) (Fig 3A). Similar to PCC, algorithm scores trended towards higher values for early onset of CHB with mean score of 11.9 versus late onset of CHB with a mean score of 10.0; however, this was not a statistically significant difference (p = .0889) (Fig 3B). However, we did see significant differences for the onset of RA and ccRCC (Fig 3C and 3D). For RA, early onset mutations had an average algorithm score of 11.8 while late onset scores had an average algorithm score of 8.5 (Fig 3C). For ccRCC, early onset mutations had an average algorithm score of 13.1 while late onset mutations had an average algorithm score of 10.1 (Fig 3D).

These data indicate that our algorithm can distinguish more pathogenic mutations from less pathogenic ones that are based on age-related onset of different VHL related cancer types. While not significant at the α-value cut-off at .05, the scores for early age of onset for both PCC and CHB trended towards higher values than the later age of onset. For ccRCC and PA, the scores for early onset versus the scores late onset were significantly higher at α-value cut off of .05. Larger patient datasets from similar studies could be used to further refine our algorithm, and determine significance for both PCC and CHB disease types. Our analysis provide significant support for the use and refinement of *in silico* evaluation of *VHL* mutations and their capacity for large scale protein dysfunction to predict pathogenic outcomes.

## Discussion and conclusions

Von Hippel-Lindau (VHL) disease is an autosomal dominant hereditary disease that causes a variety of highly vascularized tumors in patients [68,69]. While the average life expectancy is around 65 years of age, secondary conditions of tumor development such as blindness or neurological complications can be debilitating. Should these complications go undiagnosed and subsequently untreated, VHL becomes a fatal condition [1,24]. Genetic diagnosis of VHL disease provides an early detection method for clinicians to begin surveillance. Computational and biophysical approaches aimed at predicting the severity of a mutation and its deleterious consequences on the function of pVHL can contribute additional information on how the disease might progress. We have provided a multiparameteric algorithmic approach to evaluate the severity of missense mutations in *VHL*. pVHL functions as a scaffold for the creation of the E3 ubiquitin ligase complex for proper regulation of HIF; therefore, our comprehensive evaluation of pVHL misfolding and dysfunction provides a structurally and molecularly informed approach to the prediction of mutation severity.

Our approach was able to distinguish between the populations of benign and pathogenic ClinVar mutations (Fig 1D and S2B Fig). We also observed significantly higher algorithm scores for those mutations deemed high risk of ccRCC by Symphony (S3B Fig). Taken together, our multiparameteric algorithm can be used to identify pathogenic from benign mutations in pVHL.

pVHL functions as a scaffold for the assembly of the VCB-CR complex [9]. Perturbations to its secondary structure and binding capacity can have deleterious effects on the function of this complex, primarily the negative regulation of HIF under normoxia [2,13,16]. The N-terminal tail of pVHL is only present in the pVHL30 isoform, with mutations occurring in this region being mostly ranked as clinically benign [33,62]. Our algorithm scores also demonstrated significantly lower scores for mutations occurring in the N-terminus compared to the α- or β-domains of the protein (Fig 2B). The N-terminus is predicted to exist as an unstructured/random coil region; therefore, we expected lower average algorithm scores for mutations occurring in the coil regions of pVHL (Fig 2A). Finally, the N-terminus includes binding interface D, one of the five binding interfaces of pVHL, which is not known to interact with proteins crucial for the regulation of HIF [8,33,62]. Similar to the N-terminal domain and the random coil regions of pVHL, interface D has the lowest average algorithm score compared to the other binding interfaces (Fig 2C). Interface E consists of the C-terminal helix of pVHL; however, not much is known about its potential binding partners and its involvement in VHL disease [8,33,64]. Our data indicate that mutations occurring on interface E are less pathogenic, having a lower algorithm score than mutations occurring on binding surface A or B (Fig 2C). Binding surfaces A, B, and C are involved in VCB complex formation, HIF, and Cul2 binding, respectively [8,33,62]. Mutations that occur in this region are poised to interrupt the protein interactions crucial for HIF regulation, leading to tumor development. This is indicated by their higher average mutations scores of 11.5, 9.9, and 8.5 for surfaces A, B, and C, respectively (Fig 2C and S4 File). Mutations in binding interface A score significantly higher against all other interfaces, while B and C score significantly higher than the N-terminal interface D (Fig 2C and S4 File). These observations are consistent with the biological functions of these interfaces in the pathogenicity of VHL disease. Since these surfaces are involved in the formation of the E3 ubiquitin ligase complex, the higher algorithm scores are reflective of the potential dysfunction that result from mutations in these regions. pVHL functions to complex proteins together; therefore, mutations occurring on the surface of the protein, regardless of interface, should be more deleterious to overall function than mutations occurring towards the interior of the protein [34]. Using a set of defined surface and deep mutations, our algorithmic approach scored surface mutations higher than deep mutations (Fig 2D). This is in agreement with other studies which found surface mutations to be at a higher risk of developing PCC [34].

VHL is an autosomal dominant hereditary disease putting patients at a lifelong risk of tumor development. Upon spontaneous mutation of the wild-type allele in susceptible tissue types, tumor development begins. A predictive outlook for the onset of VHL related cancers could provide clinicians with a more personalized surveillance strategy when provided with a unique mutation. Using data curated from the literature, our algorithm scored missense mutations associated with an earlier age of onset for RA and ccRCC higher than those associated with a late age of onset (Fig 3C and 3D) [26,34]. While, there was a trend towards higher algorithm scores for the early age of onset of PCC and CHB this was not statistically significant at the α-value cut off of .05 (Fig 3A and 3B). Our multiparametric method scored W117 and L184, two residues identified as prone to highly destabilizing mutations, with high average scores of 12.84 and 14.83, respectively (Table 1) [29]. The approach outlined in this paper can identify mutations that are destabilizing, but this trend was not maintained for all mutations identified as VHL mutation hot spots, such as Y98 (Table 2) [33]. Our multiparametric scoring algorithm evaluates the consequences of a missense mutation on the overall stability and folding dynamics of pVHL. Pathogenic mutations with lower algorithm scores, such as Y98, may serve a more direct role in protein-protein interactions or posttranslational modification, may be missed in our algorithm. However, it is dfting to speculate that biochemical studies on clinically identified hotspots that are scored lower in our algorithm, may reveal critical residues for VHL function not previously identified.

Additional clinical data will allow us to iteratively refine our algorithm approach. For example, some same-sense mutations can cause exon skipping in VHL, like the synonymous c.414A>G, p.Pro138Pro mutation [70,71] The dysregulation of splicing creates a truncated protein product consisting of exons 1 and 3. This deleterious variant of pVHL is unable to regulate HIF expression [70,71]. For synonymous mutations such as c.414A>G, our algorithmic approach, would give this mutation an overall score of 1, as it can only alter the translation rate of the native codon. In exceptional cases as this clinical mutation, a more detailed understanding of the mechanism of exon skipping could inform future algorithmic approaches for the assessment of exon skipping risk in the *VHL* gene.

We have provided the first comprehensive multiparametric assessment of VHL missense mutations on the function of the VHL protein. Our platform provides the first steps to understand the phenotypic heterogeneity associated with missense mutations in pVHL. We anticipate that our algorithm can undergo iterative refinement as additional clinical data is made available, and the predictive capacity of our approach can be therefore be improved as additional research on VHL is available.

## Supporting information

S1 Fig Algorithm score distributions for unweighted multiparametric assessment of VHL missense mutations. **A.** Score distributions for all possible missense mutations from a SNP. A gaussian distribution was fitted to the data (red). **B.** Score distribution for the ClinVar mutation data set. A gaussian distribution was fitted to the data (red).
(TIF)

S2 Fig Distribution of benign versus pathogenic ClinVar mutations. **A.** Algorithm score distributions for the unweighted ClinVar mutation data set. **B.** Algorithm score distributions for the weighted ClinVar mutation data set. All data was fitted with a Gaussian distribution. Benign scores are in black. Pathogenic mutations are in red.
(TIF)

S3 Fig Algorithm score association to pathogenicity indictators. **A.** Scores for the ClinVar missense mutation annotation. Each dot represents a missense mutation. A * indicated a P < .05 as determined by ANOVA and Tukey HSD. **B.** Scores for ClinVar missense mutations identified as high or low risk of ccRCC by Symphony. A * indicates a P < .05 as indicated by a students t-test. All error bars represent the standard deviation. All statistics were done using GraphPad Prizm software.
(TIF)

S1 Table Phi coefficient values to test for independence of the eight parameters used to evaluate VHL missense mutations. Values were calculated in R using the unweighted algorithm scores for the ClinVar mutation data set.
(TIF)

S2 Table Chi square values of the eight parameters for their association to the pathogenicity index score of 211 ClinVar missense mutations for which a ClinVar annotation and a Symphony prediction of ccRCC were present. P-values were used to delineate the weighting strategy.
(TIF)

S1 File All missense muts. (XLSX)

S2 File ClinVar missense. (XLSX)

S3 File Cancer onset. (XLSX)

S4 File GraphPad statistics. (XLSX)

S5 File (SCR)

S6 File (PY)

S7 File (PY)

S8 File (PY)

S9 File (PY)

S10 File (PY)

S11 File (PY)

S12 File (PY)

S13 File (PY)

S14 File (SCR)

S15 File (SCR)
