# Supplementary figures and images for: Algorithmic assessment of missense mutation severity in the Von-Hippel Lindau protein
Source: PLoS One. 2020 Nov 5;15(11):e0234100. doi: 10.1371/journal.pone.0234100 (PMC7644048; doi:10.1371/journal.pone.0234100)

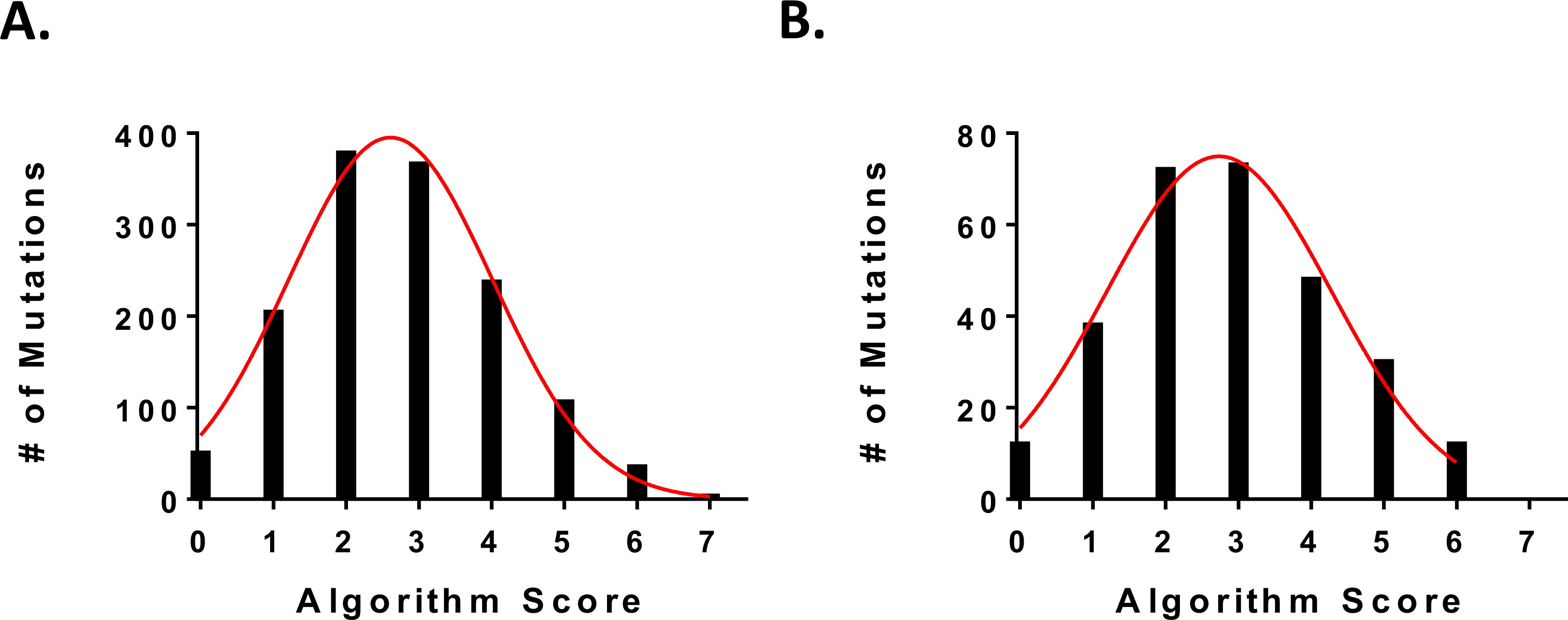

Supplement: S1 Fig — A. Score distributions for all possible missense mutations from a SNP. A gaussian distribution was fitted to the data (red). B. Score distribution for the ClinVar mutation data set. A gaussian distribution was fitted to the data (red). (TIF) [file pone.0234100.s001.tif]

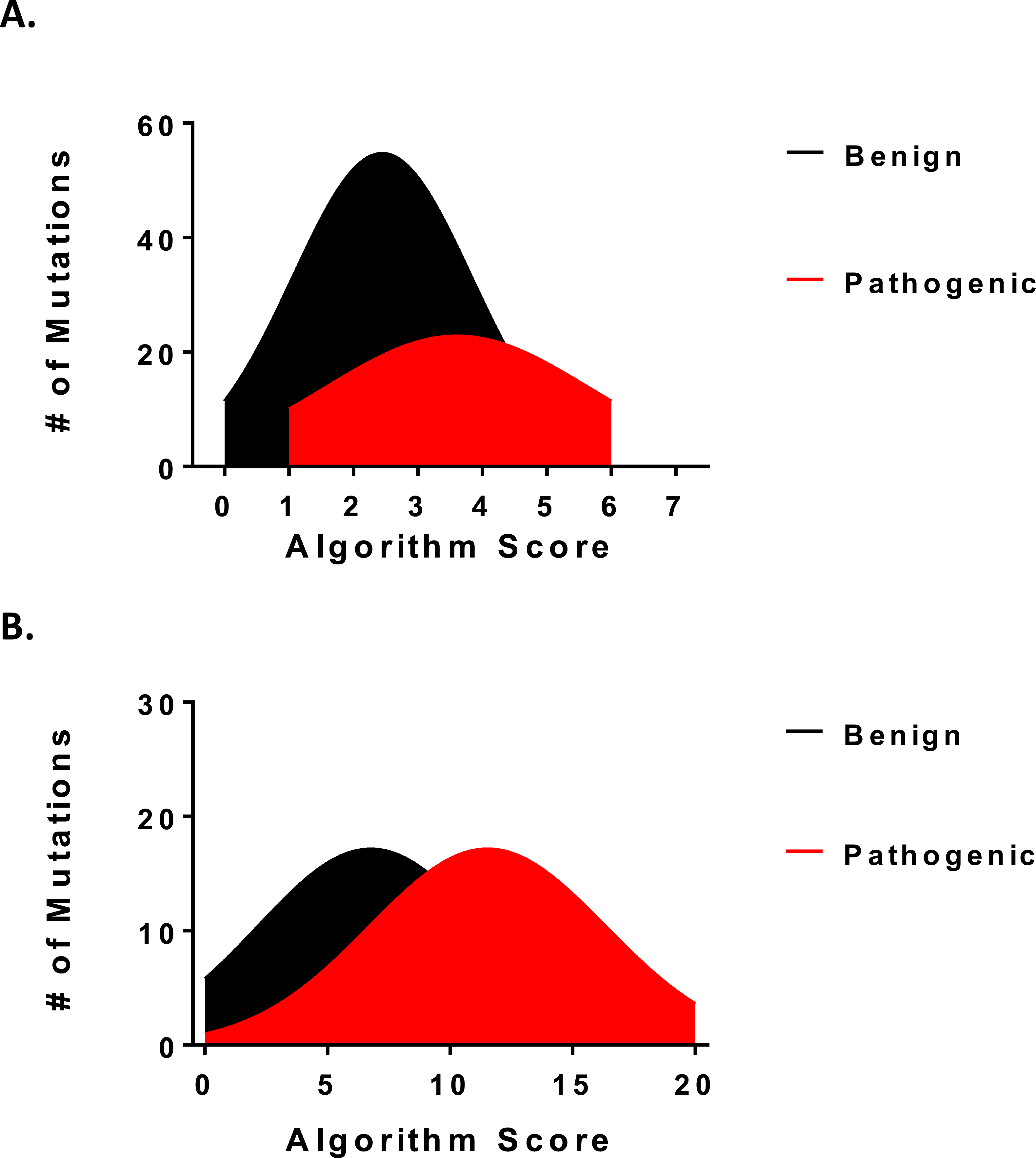

Supplement: S2 Fig — A. Algorithm score distributions for the unweighted ClinVar mutation data set. B. Algorithm score distributions for the weighted ClinVar mutation data set. All data was fitted with a Gaussian distribution. Benign scores are in black. Pathogenic mutations are in red. (TIF) [file pone.0234100.s002.tif]

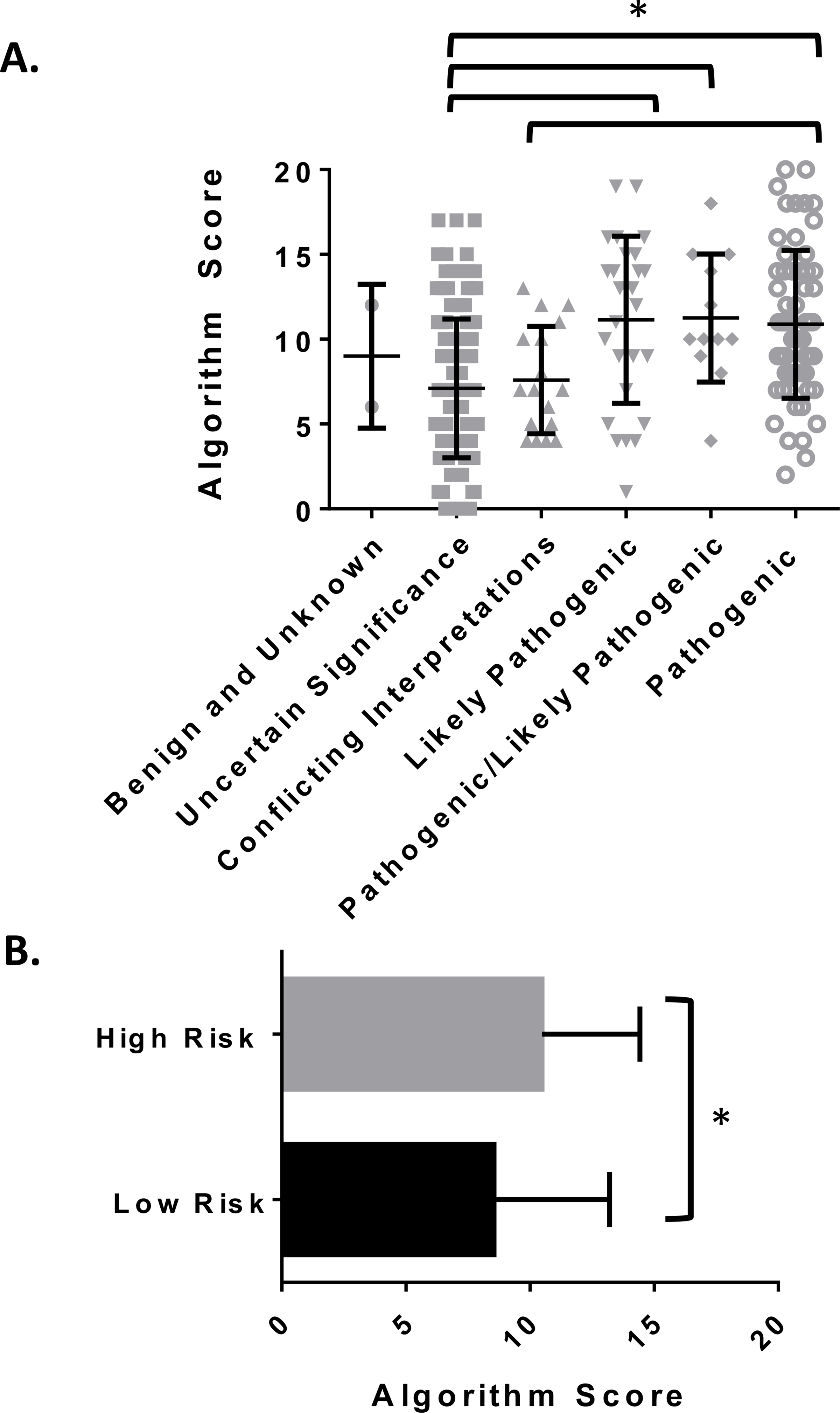

Supplement: S3 Fig — A. Scores for the ClinVar missense mutation annotation. Each dot represents a missense mutation. A * indicated a P < .05 as determined by ANOVA and Tukey HSD. B. Scores for ClinVar missense mutations identified as high or low risk of ccRCC by Symphony. A * indicates a P < .05 as indicated by a students t-test. All error bars represent the standard deviation. All statistics were done using GraphPad Prizm software. (TIF) [file pone.0234100.s003.tif]

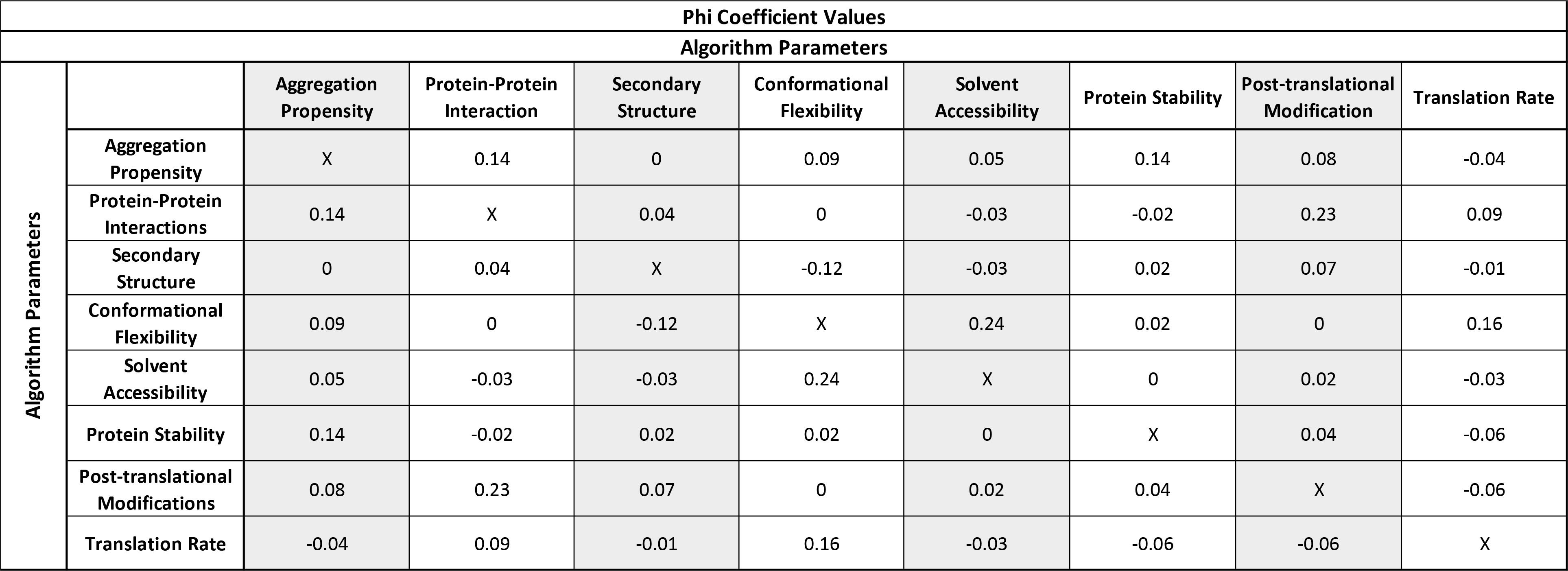

Supplement: S1 Table — Values were calculated in R using the unweighted algorithm scores for the ClinVar mutation data set. (TIF) [file pone.0234100.s004.tif]

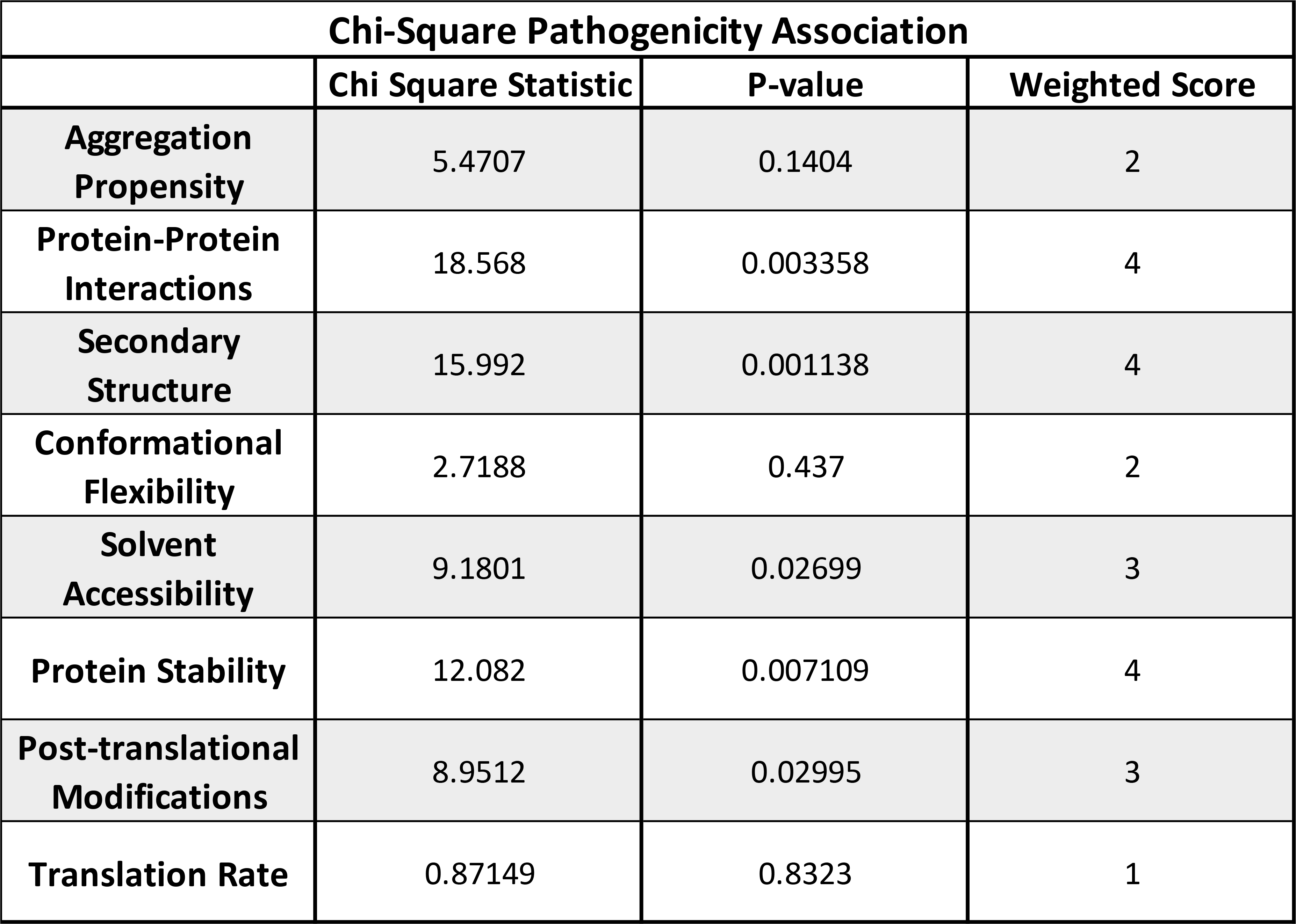

Supplement: S2 Table — P-values were used to delineate the weighting strategy. (TIF) [file pone.0234100.s005.tif]
